# Physical activity-mediated associations between perceived neighborhood social environment and depressive symptoms among Jackson Heart Study participants

**DOI:** 10.1186/s12966-020-00991-y

**Published:** 2020-07-10

**Authors:** Kosuke Tamura, Steven D. Langerman, Stephanie L. Orstad, Sam J. Neally, Marcus R. Andrews, Joniqua N. Ceasar, Mario Sims, Jae E. Lee, Tiffany M. Powell-Wiley

**Affiliations:** 1 Social Determinants of Obesity and Cardiovascular Risk Laboratory, Cardiovascular Branch, Division of Intramural Research, National Heart, Lung, and Blood Institute, National Institutes of Health, 10 Center Drive, Bethesda, MD 20892 USA; 2 Department of Medicine, Johns Hopkins University, Baltimore, MD USA; 3 Department of Medicine, Division of General Internal Medicine and Clinical Innovation, New York University Grossman School of Medicine, New York, NY USA; 4 Department of Medicine, University of Mississippi Medical Center, Jackson, MS USA; 5 Research Centers in Minority Institutions Translational Research Network Data Coordinating Center, Jackson State University, Jackson, MS USA; 6 Intramural Research Program, National Institute on Minority Health and Health Disparities, National Institutes of Health, Bethesda, MD USA

**Keywords:** Perceived neighborhood environment, Depression, African-Americans, Physical activity, mediators, Jackson heart study

## Abstract

**Background:**

Little is known about the associations between perceived neighborhood social environment (PNSE) and depressive symptoms among African Americans. Furthermore, the role of physical activity (PA) as a mediator of this association has not been investigated. The two-fold objectives of this study, therefore, were (1) to examine the associations between PNSE and depressive symptoms among African Americans, and (2) to test the degree to which these associations were mediated by total PA.

**Methods:**

We used baseline data from the Jackson Heart Study (JHS), a single-site, prospective, community-based study of African-American adults (*n* = 2209) recruited from Jackson, Mississippi. PNSE variables included scores for neighborhood violence (i.e., higher score = more violence), problems (higher score = more problems), and social cohesion (higher score = more cohesion). Depressive symptoms were measured by the 20-item Center for Epidemiological Studies Depression (CES-D) score. First, multilevel modeling, controlling for census tract clustering effects, was used to estimate associations between each PNSE variable and CES-D score, adjusting for covariates, including demographic, health-related, and population density. Second, validated, self-reported total PA, based on active living, sport, and home indices, was tested as the mediator. Multivariable linear regressions with bootstrap-generated 95% bias-corrected confidence intervals (BC CIs) were estimated to test for significant unstandardized indirect effects, controlling for all covariates.

**Results:**

Our participants were 64.2% female with a mean age of 52.6 (SD = 12.2) and a mean CES-D score of 10.8 (SD = 8.1). In the fully-adjusted model, neighborhood violence and problems were positively related to depressive symptoms (B = 3.59, 95%CI = 0.93, 6.26, and B = 3.06, 95%CI = 1.19, 4.93, respectively). Neighborhood violence and problems were also indirectly related to depressive symptoms via total PA (B = 0.26, 95%BC CI = 0.05, 0.55; and B = 0.15, 95%BC CI = 0.02, 0.34, respectively). Social cohesion was neither directly nor indirectly related to depressive symptoms.

**Conclusions:**

We found that higher levels of perceived neighborhood problems and violence were directly and positively associated with depressive symptoms. These associations may be explained in part by lower total PA levels. Future interventions to reduce depressive symptoms attributed to neighborhood features should consider emphasizing built environment features that facilitate PA increases in conjunction with community efforts to reduce neighborhood violence and problems.

## Introduction

Depressive symptoms (e.g., loss of interest in typical activities, difficulty concentrating, psychomotor changes) represent a major public health problem in the U.S., with rates of clinically significant depression exceeding 8% (approximately 1 in 12) in a representative U.S. adult population from the 2013–2016 National Health and Nutrition Examination Survey (NHANES) [1]. Depressive disorders are also one of the leading causes of disability in the U.S., and are responsible for more years lost to disability than any other condition aside from low back pain [2]. In addition, depressive symptoms are associated with an increase in all-cause and cardiovascular disease-related mortality [3–5], as well as a significantly increased risk for chronic diseases such as diabetes [6].

The determinants of depressive symptoms are multifactorial [7], and could be impacted by areal or neighborhood socioeconomic deprivation [8] (often measured by the neighborhood deprivation index [9]). More specific features of neighborhood-level characteristics, such as the perceived neighborhood social environment (PNSE), may influence depressive symptoms [10]. This has increasingly led researchers to analyze depression within a social ecological framework [11, 12], and to identify associations of various domains of neighborhood social environment (such as violence, problems, and social cohesion) with depression [13–15]. Typically, violence encompasses behaviors such as fighting and robbery [15]. Neighborhood problems refer to issues like trash and litter [13] or elements of neighborhood disorder such as vandalism [14]. Neighborhood social cohesion includes features such as trust between neighbors [14, 16] and willingness of people to work together [13]. One study used cross-sectional data from 5943 participants in the Multi-Ethnic Study of Atherosclerosis (MESA) cohort to examine the relationship between neighborhood problems and depression, and found that individuals with fewer perceived neighborhood problems had lower depressive symptoms scores [14]. Another study using MESA data demonstrated that lower levels of neighborhood social cohesion were associated with more depressive symptoms [15]. A third cross-sectional study of African-American and white residents in Baltimore, MD found that perceptions of severe problems in the neighborhood compared to perceptions of few or no problems were associated with greater odds of depression, while perceptions of greater community cohesion were associated with lower odds of depression [13]. Furthermore, a previous study among British older adults demonstrated that greater personal sense of control and quality friendships mediated the negative association between neighborhood social cohesion and depressive symptoms [17]. Another study found that the link between greater urbanicity and depressive symptoms among U.S. older adults were mediated through physical activity (PA) [18]. However, the possible role of PA as a mediator of the associations between PNSE and depressive symptoms among African Americans has not previously been investigated.

In addition to being related to PNSE [19], PA is a well-demonstrated behavioral risk factor for depression [20–23]. Studies have found that higher levels of PA are related to lower depression levels [20]. For instance, one cross-sectional study of mental health among 36,984 Canadians found that 780,000 cases of mental disorders in Canada are potentially influenced by physical inactivity [21]. Another cross-sectional study of 1947 adults in Alameda County, in California found that increased physical activity reduces both prevalent and incident depression [22]. A study of 3403 Finnish adults indicated that those who performed regular exercise experienced less depression, more positive health and fitness perceptions, and stronger feelings of social cohesion than those who performed less frequent exercise [23].

There are few studies which have investigated PA as a mediator of the relationship between neighborhood environment and depressive symptoms. A recent study of 909 Chinese seniors found that physical activity may act as a mediator for the relationship between built environment and depression, with increased neighborhood connectivity and pedestrian infrastructure positively influencing depressive symptoms [24]. However, this study emphasized objective built environment rather than PNSE [24]. As such, no study to date has investigated whether associations between PNSE and depressive symptoms were mediated by PA. In particular, no study has investigated the impact of this relationship among African-American adults in the U.S.

Understanding the factors influencing depression in African Americans is important, as disparities in depression outcomes persist in this population. Specifically, African Americans are more likely to experience recurrent major depressive episodes (odds ratio [OR] = 1.55, *P* < 0.01) and less likely to receive guideline-concordant care (OR = 0.55; *P* < 0.01), compared to whites [25]. Furthermore, depressive symptoms are underreported among African Americans due to barriers to seeking treatment such as social stigma [26, 27]. Our study, therefore, examined associations between neighborhood social environment features and depressive symptoms among African-American adults in Jackson, Mississippi, using the Jackson Heart Study (JHS). The primary aim of our study was to analyze associations between each neighborhood social environment feature (i.e., violence, problems, and social cohesion) and depressive symptoms among JHS participants, and these associations were stratified by age and sex. Our secondary aim was to test for the degree to which associations between each PNSE and depressive symptoms are mediated by total PA.

## Methods

### Study design and study participants

For this cross-sectional analysis, we used data from the JHS, a single-site, community-based cohort study of African-American adults. The study cohort comprises 5301 individuals from the tri-county (Hinds, Madison, and Rankin) area of the Jackson, Mississippi metropolitan area. The JHS is a study of genetic and environmental risk factors for cardiovascular disease (CVD) among African Americans, as described previously [28–30]. Our study data were drawn exclusively from JHS Exam 1, which was conducted between 2000 and 2004. Of the 5301 adults in the JHS cohort, 1889 were excluded from our analysis because they did not complete depression screening questionnaires. Another 202 were removed from our sample due to missing individual-level data (e.g., education, smoking, total PA, etc.) and 15 were removed due to missing data on prior disease or disability. Those missing psychosocial measures such as lifetime discrimination and chronic stress were removed (*n* = 984). Additional participants (*n* = 2) were removed due to missing neighborhood data, which resulted in our total analytic sample of 2209. The study was approved by the institutional review boards of the University of Mississippi Medical Center, Jackson State University, and Tougaloo College. All participants provided informed consent. The National Institutes of Health Office of Human Subjects Research Protections approved our current study.

### Depressive symptoms

To measure depressive symptoms, participants responded to the 20-item Center for Epidemiologic Studies - Depression (CES-D) scale [31, 32]. Respondents were prompted to indicate the frequency with which they experienced a series of different emotional states throughout the previous week, resulting in a score between 0 and 3 for each of the 20 questions and a possible total score range of 0–60. Greater levels of depressive symptoms consistent with depression has been defined by a CES-D score of≥16 for the JHS participants [33] and validated previously [34]. In this study, the CES-D score was treated as a continuous variable. This measure had good internal reliability in the JHS (α =0.82) [35].

### Perceived neighborhood social environment (PNSE)

PNSE was classified into three categories: neighborhood violence, problems and social cohesion. These scales were obtained based on principal component analysis with Promax oblique rotation [36]. The neighborhood violence scale included 5 items which asked about topics including neighborhood fighting, sexual assault, and robbery. Respondents were asked to rate the frequency of each, with a score ranging from 1 (never) to 4 (often). The neighborhood problems scale included 6 items about topics such as neighborhood noise, lack of park access, and litter, with scores ranging from 1 (not really a problem) to 4 (a very serious problem). The neighborhood social cohesion scale was composed of 5 items which inquired about topics such as trust in neighbors and willingness to help neighbors, with scores for each item ranging from 1 (strongly disagree) to 4 (strongly agree). The three scales displayed acceptable internal consistency, with Cronbach’s alpha 0.80 (violence), 0.76 (problems), and 0.77 (social cohesion) [36]. Higher neighborhood violence and neighborhood problems scores indicated a less favorable perception of the neighborhood; in contrast, a higher neighborhood social cohesion score indicated a more favorable perception of one’s neighborhood. Using unconditional empirical Bayes estimation adjusting for age and sex, the responses of each construct for participants were aggregated to the census tract level to improve estimates for census tracts with fewer participants [37]. The participants resided across a total of 112 census tracts with a median of 19 participants per tract [36].

### Mediator

PA was assessed at the JHS baseline examination through the JHS Physical Activity (JPAC) Survey [38]. This 30-item questionnaire evaluates PA within three domains: active living, home/life, and sport/exercise activities. Scores for each domain range from 0 to 4, with a lower score corresponding to less PA. The total PA score is then calculated by adding together the scores from the three domains. This survey was adapted from the Baecke PA questionnaire from the Atherosclerosis Risk in Communities Study, and was tested against previously validated waist-worn accelerometer data [38, 39]. This total PA score was treated as a mediator.

### Covariates

We controlled for demographic and health-related covariates that could confound associations between perceived environment and CES-D scores. Individual-level covariates included age in years [17], sex (males/females) [17], high school graduation status (Y/N) [24], household income (≥ $50,000, <$50,000, or not reported) [17], BMI (body mass index, calculated from measured weight [kilograms] divided by measured height [meters] squared) [40], total PA (also used in the mediation analysis), smoking status (Y/N) [40], alcohol use (Y/N) [40], walking limitation (Y/N) [41], and history of significant medical condition (Y/N; heart attacks, cancer, stroke, diabetes, kidney problems, lung disease, or poor blood circulation) [24, 42]. We also controlled for psychosocial factors that may confound associations between PNSE and depressive symptoms. These included lifetime discrimination [43], daily discrimination [43], burden of lifetime discrimination [43], chronic stress (i.e., ongoing stressful condition over 12 months) [44], and weekly stress (i.e., minor stressful events during the past week) [44], none of which were highly correlated (r = 0.16 to 0.38). In particular, those residing in poor neighborhood conditions may be under prolonged stressful situations, and may not have access to resources to manage stressors, which may lead to greater risk for depression [8]. These variables were based on scale scores which were subsequently standardized by computing *z* scores [45]. We also controlled for population density (1000 people/km^2^), a neighborhood-level covariate known to associate with both PNSE and depression [46].

### Statistical analysis

We summarized all the study variables in Table 1 and stratified based on a median of each neighborhood social environment variable. Selected key participants’ characteristics included and not included in the analyses were presented in supplemental file. We used multilevel modeling (i.e., PROC MIXED in SAS) to account for the two-level nested data structure (i.e., individuals were nested within census tracts). Subsequently, for the first aim, we examined the association between each perceived social environment variable (neighborhood violence, problems, social cohesion) and depressive symptoms in age-adjusted and fully-adjusted models. Fully-adjusted models controlled for age, sex, high school graduation, household income, BMI, total PA, smoking status, alcohol use, walking limitation, history of a medical condition, lifetime discrimination, daily discrimination, burden of lifetime discrimination, chronic stress, weekly stress, and population density. All analyses were conducted using SAS version 9.4 (SAS® Institute, Inc., Cary, NC). We also explored age- and sex-moderated effects on the associations between each social environment variable and depressive symptoms (Supplemental tables and figures).

**Table 1:** Individual characteristics, health-related factors, psychosocial factors, and neighborhood environmental factors (*n* = 2209)

	Overall (***n*** = 2209)	Neighborhood Violence^**a**^		***P***	Neighborhood Problems^**a**^		***P***	Neighborhood Social Cohesion^**a**^		***P***
	n (%)	High ***n*** = 1116	Low ***n*** = 1093		High ***n*** = 1157	Low ***n*** = 1052		High ***n*** = 1166	Low ***n*** = 1043	
**Individual characteristics**										
Age (years), M (±SD)	52.64 (±12.20)	54.97 (±12.36)	50.27 (±11.57)	<.0001	54.96 (±12.39)	50.10 (±11.47)	<.0001	52.11 (±12.00)	53.25 (±12.40)	0.0283
Female	1418 (64.19)	742 (66.49)	417 (38.15)	0.0230	773 (66.81)	645 (61.31)	0.0071	739 (63.38)	679 (65.10)	0.3995
High School Graduate				<.0001			<.0001			<.0001
Yes	1959 (88.68)	938 (84.05)	1021 (93.41)		969 (83.75)	990 (94.11)		1072 (91.94)	887 (85.04)	
No	250 (11.32)	178 (15.95)	72 (6.59)		188 (16.25)	62 (5.89)		94 (8.06)	156 (14.96)	
Income				<.0001			<.0001			<.0001
≥ $50,000	783 (35.45)	289 (25.90)	494 (45.20)		286 (24.72)	497 (47.24)		499 (42.80)	284 (27.23)	
< $50,000	1140 (51.61)	689 (61.74)	451 (41.26)		730 (63.09)	410 (38.97)		515 (44.17)	625 (59.92)	
Not reported	286 (12.95)	138 (12.37)	148 (13.54)		141 (12.19)	145 (13.78)		152 (13.04)	134 (12.85)	
**Health-related factors**										
Body Mass Index, M (±SD)	31.83 (±7.18)	32.10 (±7.40)	31.56 (±6.95)	0.0780	31.90 (±7.32)	31.76 (±7.03)	0.6354	31.60 (±6.90)	32.10 (±7.48)	0.1045
Total Physical Activity	6.76 (±1.96)	6.48 (±1.96)	7.04 (±1.93)	<.0001	6.54 (±1.98)	7.00 (±1.91)	<.0001	6.93 (±1.97)	6.56 (±1.93)	<.0001
Current smoker	250 (11.32)	144 (12.90)	106 (9.70)	0.0174	150 (12.96)	100 (9.51)	0.0104	121 (10.38)	129 (12.37)	0.1404
Alcohol drinker^b^	1093 (49.48)	505 (45.25)	588 (53.80)	<.0001	522 (45.12)	571 (54.28)	<.0001	605 (51.89)	488 (46.79)	0.0167
Disabled from walking	95 (4.30)	58 (5.20)	37 (3.39)	0.0358	62 (5.36)	33 (3.14)	0.0101	50 (4.29)	45 (4.31)	0.9757
History of Medical Condition	721 (32.64)	417 (37.37)	304 (27.81)	<.0001	438 (37.86)	283 (26.90)	<.0001	332 (28.47)	389 (37.30)	<.0001
Depressive Symptoms based on CES-D score	10.79 (±8.05)	11.89 (±8.47)	9.66 (±7.43)	<.0001	11.89 (±8.36)	9.58 (±7.52)	<.0001	9.92 (±7.60)	11.76 (±8.43)	<.0001
**Psychosocial factors, M (±SD)**										
Lifetime discrimination	3.55 (±1.89)	3.40 (±1.87)	3.70 (±1.91)	0.0002	3.41 (±1.88)	3.70 (±1.90)	0.0004	3.62 (±1.88)	3.47 (±1.91)	0.0523
Daily discrimination	2.20 (±0.99)	2.20 (±1.05)	2.19 (±0.93)	0.8077	2.21 (±1.04)	2.19 (±0.94)	0.7354	2.20 (±0.96)	2.20 (±1.03)	0.9742
Burden of lifetime discrimination	2.35 (±0.77)	2.38 (±0.78)	2.32 (±0.76)	0.0785	2.38 (±0.78)	2.33 (±0.76)	0.1498	2.35 (±0.77)	2.36 (±0.77)	0.6732
Chronic stress	5.64 (±4.45)	5.87 (±4.69)	5.40 (±4.17)	0.0120	5.86 (±4.70)	5.39 (±4.14)	0.0112	5.28 (±4.18)	6.04 (±4.70)	<.0001
Weekly stress	87.22 (±82.25)	90.33 (±86.44)	84.03 (±77.65)	0.0716	90.77 (±86.08)	83.31 (±77.67)	0.0325	81.30 (±76.67)	93.84 (±87.63)	0.0004
**Neighborhood Environment at the census tract level, M (±SD)**										
*Perceived neighborhood social environment*^*c*^										
Violence	1.25 (±0.12)	1.35 (±0.09)	1.15 (±0.05)	<.0001	1.34 (±0.09)	1.15 (±0.05)	<.0001	1.19 (±0.07)	1.32 (±0.13)	<.0001
Problems	1.55 (±0.19)	1.69 (±0.13)	1.40 (±0.10)	<.0001	1.69 (±0.12)	1.38 (±0.08)	<.0001	1.44 (±0.12)	1.66 (±0.18)	<.0001
Social cohesion	3.02 (±0.12)	2.95 (±0.12)	3.09 (±0.08)	<.0001	2.95 (±0.11)	3.09 (±0.08)	<.0001	3.11 (±0.06)	2.92 (±0.08)	<.0001
*Objective built environment*^*d*^										
Population density (people/km^2^)	791.61 (±487.82)	1012.09 (±420.60)	566.50 (±447.36)	<.0001	1088.86 (±365.89)	464.69 (±385.26)	<.0001	620.88 (±463.75)	982.48 (±441.26)	<.0001

***Note***

^a^Based on the median

^b^Alcohol consumption in the past 12 months

^c^Each perceived social environment variable was aggregated to census-tract level based on unconditional empirical Bayes estimation adjusting for age and sex

^d^Population density was measured around 1 mile from participant’s residence

*P*-values were based on t-tests for continuous variables and chi-square tests for categorical variables.

**Fig. 1:**
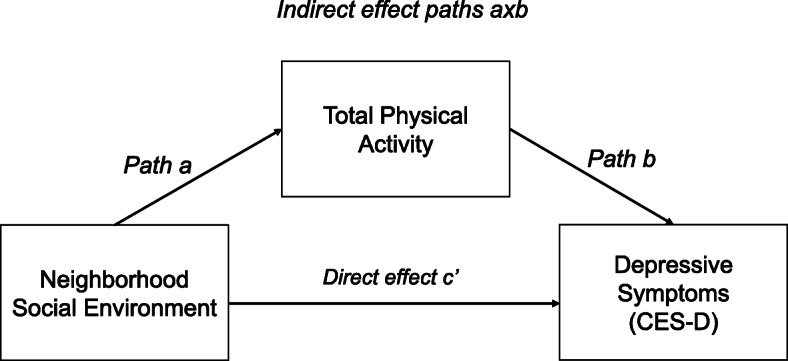
Conceptual model of mediation analysis. Note: This model examined indirect (paths axb) and direct (path c’) relationships between neighborhood social environment and depressive symptoms among Jackson Heart Study participants

For the secondary aim (i.e., mediation analysis), we employed bootstrap resampling (*k* = 5000) with 95% bias-corrected confidence intervals (BC CIs) of the indirect effects to test for mediation through the total PA (see the conceptual model in Fig. 1) on the associations between PNSE and depressive symptoms using PROCESS© v3.3 for SAS 9.4 (SAS® Institute, Inc., Cary, NC) [47]. Statistically significant mediation was determined by a BC CI without inclusion of zero. The bootstrapping resampling approach was used for testing mediation because it yields an inferential test of indirect effects of the exposures on the outcomes via the mediators, reduces type I errors, has greater statistical power than traditional causal steps approach for mediation tests, and does not need a normal sampling distribution [47].

## Results

### Sample characteristics

Participant characteristics are shown in Table 1. Participants’ mean age was 52.6 years (SD ± 12.2). A majority (about 64%) of participants were female, and most were high school graduates. Over half had income levels below $50,000 per year. The mean BMI of the participants was 31.8, and the mean total PA score was 6.8 (SD ± 1.96). Nearly half (49.5%) reported alcohol use in the past 12 months, while some (11.3%) reported cigarette smoking. Participants had a mean depression score (CES-D depressive symptoms score) of 10.8.

Participants perceiving higher levels of neighborhood violence were more likely to be older (*p* < .0001), be female (*p* < .03), have less than a high school diploma (*p* < .0001), be low-income (*p* < .0001), be physically inactive (*p* < .0001), be current smokers (*p* < .02), ne non-alcohol drinkers (*p* < .0001), be disabled from walking (*p* < .04), or have a history of a medical condition (*p* < .0001), compared to individuals perceiving lower neighborhood violence (Table 1). Furthermore, participants perceiving higher levels of neighborhood violence had significantly lower scores for lifetime discrimination (*p* = 0.0002). The patterns of association of socio-demographics, health-related factors, and psychosocial factors with neighborhood violence appeared to be similar to associations of these factors with high neighborhood problems and low neighborhood social cohesion (Table 1).

### Associations between PNSE and depressive symptoms

In an age-adjusted model, a one-unit increase in neighborhood violence and problems was associated with a 10.6 and a 8.0 point higher in the depressive symptoms score, respectively. In an age-adjusted model considering social cohesion, however, a one-unit increase in social cohesion was associated with an 9.1 point lower depressive symptoms score (data not shown). In a fully-adjusted model, a one-unit increase in neighborhood violence or problems was associated with a 3.59 and 3.06 point higher depressive symptoms score respectively (*p* < 0.01), which attenuated from the age-adjusted model (Table 2). There was no significant relationship between neighborhood social cohesion and depressive symptoms score in the fully-adjusted model.

**Table 2:** Associations between neighborhood social environment and depressive symptoms scores controlling for covariates among Jackson Heart Study participants (*n* = 2209)

	Neighborhood Violence		Neighborhood Problems		Neighborhood Social Cohesion	
	B (SE)	95% C.I.	B (SE)	95% C.I.	B (SE)	95% C.I.
Intercept	7.69 (2.06)***	3.61, 11.78	7.71 (1.86)***	4.02, 11.41	17.78 (4.19)***	9.49, 26.07
**Neighborhood Social Environment**^**a**^	3.59 (1.34)**	0.93, 6.26	3.06 (0.94)**	1.19, 4.93	−1.96 (1.30)	−4.53, 0.61
**Individual Characteristics**						
Age (years)	0.00 (0.01)	−0.02, 0.03	0.00 (0.01)	−0.03, 0.03	0.01 (0.01)	−0.02, 0.03
Sex						
Female	Ref.		Ref.		Ref.	
Male	−1.13 (0.31)***	−1.74, −0.51	− 1.13 (0.31)***	− 1.74, − 0.51	−1.11 (0.31)***	− 1.72, − 0.49
High School Graduate						
No	Ref.		Ref.		Ref.	
Yes	−1.98 (0.47)***	−2.93, − 1.02	−1.97 (0.47)***	− 2.92, − 1.02	−2.02 (0.48)***	− 2.97, − 1.07
Income						
≥ $50,000	Ref.		Ref.		Ref.	
< $50,000	1.77 (0.33)***	1.11, 2.43	1.72 (0.33)***	1.06, 2.38	1.83 (0.33)***	1.17, 2.48
Not reported	1.06 (0.46)*	0.14, 1.98	1.05 (0.46)*	0.13, 1.97	1.11 (0.46)*	0.19, 2.03
**Health-Related Factors**						
Body Mass Index, M (±SD)	0.01 (0.02)	−0.03, 0.05	0.01 (0.02)	−0.02, 0.05	0.01 (0.02)	−0.02, 0.05
Total Physical Activity	−0.23 (0.07)**	− 0.38, − 0.09	−0.23 (0.07)**	− 0.38, − 0.09	−0.24 (0.07)**	− 0.39, − 0.10
Current smoker						
No	Ref.		Ref.		Ref.	
Yes	0.89 (0.45)	−0.01, 1.80	0.89 (0.45)	−0.01, 1.79	0.92 (0.45)*	0.02, 1.82
Alcohol consumption						
No	Ref.		Ref.		Ref.	
Yes	0.19 (0.30)	−0.40, 0.77	0.19 (0.30)	−0.39, 0.78	0.16 (0.30)	−0.42, 0.75
Disabled from walking						
No	Ref.		Ref.		Ref.	
Yes	1.46 (0.70)*	0.03, 2.88	1.44 (0.70)*	0.02, 2.86	1.44 (0.70)*	0.01, 2.86
History of Medical Condition						
No	Ref.		Ref.		Ref.	
Yes	0.93 (0.31)**	0.32, 1.54	0.94 (0.31)**	0.32, 1.55	0.94 (0.31)**	0.32, 1.55
**Psychosocial Factors**^**b**^						
Lifetime discrimination	−0.47 (0.18)**	− 0.83, − 0.11	− 0.46 (0.18)*	− 0.82, − 0.11	− 0.49 (0.18)**	− 0.85, − 0.14
Daily discrimination	0.72 (0.16)***	0.40, 1.04	0.71 (0.16)***	0.39, 1.03	0.72 (0.16)***	0.40, 1.04
Burden of lifetime discrimination	0.79 (0.16)***	0.47, 1.12	0.80 (0.16)***	0.47, 1.12	0.80 (0.17)***	0.48, 1.12
Chronic stress	1.32 (0.16)***	1.00, 1.64	1.32 (0.16)***	1.00, 1.63	1.33 (0.16)***	1.01, 1.66
Weekly stress	2.91 (0.15)***	2.61, 3.21	2.90 (0.15)***	2.60, 3.21	2.91 (0.15)***	2.61, 3.21
**Built Environment**^**c**^						
Population density (1000 people/km^2^)	−0.15 (0.33)	− 0.81, 0.50	− 0.35 (0.35)	− 1.04, 0.34	0.06 (0.32)	− 0.57, 0.69

***Note***

^a^Each perceived social environment variable was based on unconditional empirical Bayes estimation adjusting for age and sex

^b^Based on scale sores which were standardized by computing z scores with mean zero and one standard deviation

^c^Population density was measured around one mile from participant’s residence

*P*-values

*p < 0.05

**p < 0.01

***p < 0.001

### Associations between individual, health-related, and objective built environment variables and depressive symptoms

With respect to covariates, being male (compared to female), having a high school degree, and having higher total PA were negatively associated with depressive symptoms (Table 2). In contrast, having an annual income less than $50,000, being disabled from walking, and having a history of a medical condition were positively associated with depressive symptoms. Higher standardized scores for daily discrimination, burden of lifetime discrimination, chronic stress, and weekly stress were significantly associated with lower depressive symptoms scores. However, age, BMI, smoking status, alcohol consumption, and population density were not associated with depressive symptoms. The patterns of these covariates appeared to be similar when evaluating neighborhood problems and social cohesion separately in relation to depressive symptoms.

### Associations between PNSE and depressive symptoms, stratified by age and sex

There was no significant interaction by age and sex when we tested effect modification; however, some JHS analyses are stratified by age or sex [48–50]. As such, we also stratified our fully-adjusted model by age (< 55 vs. ≥ 55) and sex (male vs. female), finding that neighborhood violence was significantly associated with depressive symptoms only in females under 55 (B = 5.74; 95% CI = 0.85, 10.63) (Supplemental Table 1 and Supplemental Figure 1). Similarly, neighborhood problems were also associated with depressive symptoms only among females under 55 (B = 4.80; 95% CI = 1.44, 8.16; Supplemental Table 2 and Supplemental Figure 2). No significant findings for neighborhood social cohesion associated with depressive symptoms were observed Supplemental Table 3 and Supplemental Figure 3).

### Associations between perceived neighborhood social environment and depressive symptoms, mediated by total PA

**Fig. 2:**
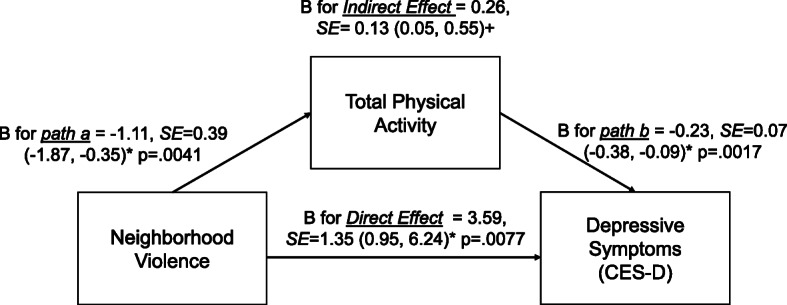
Indirect and direct relationships between neighborhood violence and depressive symptoms mediated through total physical activity. Note: *95% Confidence Intervals (95% CIs), +95% bias-corrected confidence  intervals (95% BC CIs)

**Fig. 3:**
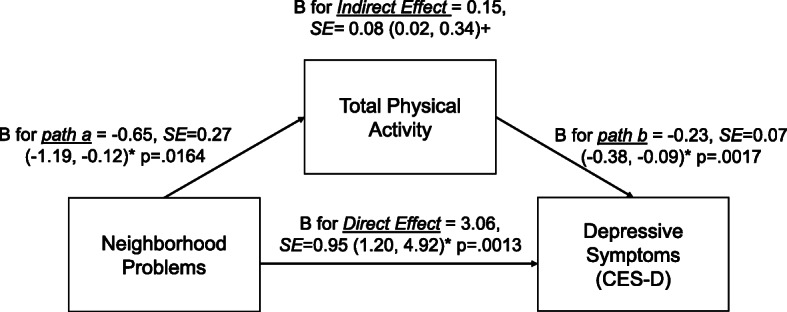
Indirect and direct relationships between neighborhood problems and depressive symptoms mediated through total physical activity. Note: *95% Confidence Intervals (95% CIs), +95% bias-corrected confidence intervals (95% BC CIs)

**Fig. 4:**
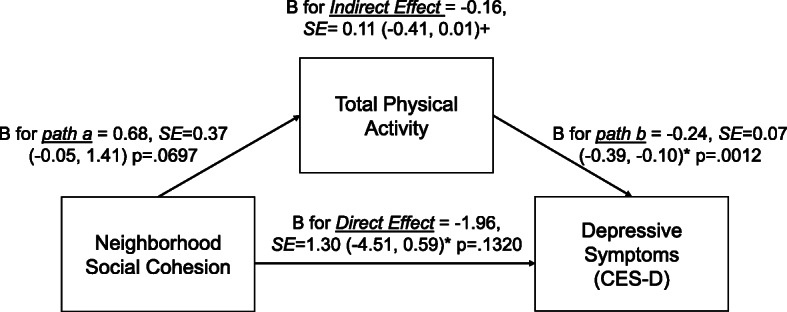
Indirect and direct relationships between neighborhood cohesion and depressive symptoms mediated through total physical activity. Note: *95% Confidence Intervals (95% CIs), +95% bias-corrected confidence intervals (95% BC CIs)

Neighborhood violence and problems were indirectly associated with depressive symptoms via total PA (B = 0.26, 95%BC CI = 0.05, 0.55; and B = 0.15, 95%BC CI = 0.02, 0.34, respectively) (Figs. 2 and 3). That is, higher violence and problems in the neighborhood were associated with lower total PA. In turn, lower total PA was associated with higher depressive symptoms. Neighborhood violence and problems were also directly related to depressive symptoms (*p* < .05). Yet, neighborhood social cohesion was not indirectly or directly related to depressive symptoms (Fig. 4). In addition, mediation analyses which excluded individuals who were disabled from walking (*n* = 95; analytic sample = 2114) were performed (Supplemental Table 4). The results were similar to those of the original analyses (*n* = 2209). Lastly, to better understand each PA domain (active living, home/life, and sport/exercise activities), we performed a sensitivity mediation analysis to examine associations between PNSE factors and depressive symptoms through each PA domain (Supplemental Tables 5, 6 and 7). The results indicated that significant indirect mediation was driven by active living and sport/exercise activities, but not home/life activities.

## Discussion

In this study of over 2000 African Americans from the JHS, we examined associations between each PNSE and depressive symptoms, while accounting for all covariates. We found that perceived neighborhood violence and problems were positively associated with depressive symptoms, but neighborhood social cohesion was not. Further, mediation analysis revealed that higher levels of neighborhood violence and problems were indirectly associated with depressive symptoms via total PA. No direct or indirect associations were found for neighborhood social cohesion. Based on these findings, future policy interventions should focus on changes in social and built environment (e.g., crime rate, neighborhood safety, and activity-friendly environment [51]) that may improve perceptions of neighborhood violence and problems.

Our study adds to the literature by demonstrating that perceived neighborhood violence is positively associated with depressive symptoms, a relationship which has not previously been investigated in a large sample of African-American adults. Studies in other populations, however, have generated similar findings. For example, one cross-sectional study of 2963 participants from Multi-Ethnic Study of Atherosclerosis (MESA) cohort found that individuals with perceived neighborhood violence scores in the highest quartile had CES-D scores 2.82 points higher than individuals with neighborhood violence scores in the lowest quartile [15]. Another study of 150 adult caregivers of children with asthma found that fear of neighborhood violence was positively associated with depression [52]. A possible explanation for the positive association between neighborhood violence and depressive symptoms is that neighborhood violence may lead to perceptions of neighborhood disorder, which may lead to psychological distress and depression [53].

Our study also demonstrates that neighborhood problems (i.e., based on the score created using principal component analysis by using items on noise and traffic; Supplemental File 1) are positively associated with depressive symptoms. A richer understanding of these associations will allow policymakers and civil engineers to better address these issues. Prior research investigating this relationship has produced similar results. For instance, one study of 1408 white and African Americans living in Baltimore, Maryland, assessed the association between self-reported neighborhood problems (e.g., insufficient garbage collection, limited street lighting, poor public transit) and depression, as defined by a Patient Health Questionnaire score ≥ 5. The authors found that African-Americans with severe problems in their communities had higher odds of depression than those without severe problems in their communities [13]. It may be that neighborhood problems (e.g., litter, physical disorder) and violence (e.g., robbery, assault) may cluster together and may operate the same pathway to link to depressive symptoms. Future research should investigate differences in neighborhood problems and violence and how these similar but different constructs play a key role for the determinants of depressive symptoms.

We found no association between neighborhood social cohesion and depressive symptoms in the current study. Research on other racial groups has found significant inverse associations between social cohesion and depressive symptoms [13–15]. For instance, one study found that individuals with social cohesion scores in the lowest quartile had higher depressive symptoms scores, compared to individuals in the highest quartile [15]. Another study using white and African American adults demonstrated that perceived neighborhood social cohesion associated with lower odds of depression in white residents, but not in African-American residents [13]. This race-specific association is an important addition to the literature and may inform future interventions and policy.

This study may be the first to test the mediating role of PA on the associations between PNSE and depressive symptoms among African Americans. Elucidating the indirect association of PA levels with depressive symptoms is particularly important, especially in light of the evidence supporting a role of PA in mitigating such symptoms [21–23]. Our findings of direct and indirect associations of higher neighborhood violence and problems with depressive symptoms were somewhat consistent with previous research on the associations between environmental factors and depression which were mediated by PA among older adults in the U.S. [18]. One study used the participants from the longitudinal National Social Life, Health and Aging Project to test for mediating effects of PA on the associations of urbanicity on depression [18]. The authors found that higher urbanicity is linked to physical inactivity, which resulted in increased depression [18]. Despite differences in neighborhood characteristics, exposures to negative social and environmental features (i.e., violence, problems, or urbanicity [18]) may be associated with lower PA, in turn, could lead to higher depressive symptoms. These results need to be interpreted cautiously. The sensitivity mediation analysis revealed that active living and sport/exercise activities may be a driving force of total PA (not home/life activities). However, a recent prospective study showed that a higher neighborhood deprivation index was negatively associated with PA (exercise) time, but positively associated with non-exercise PA (e.g., house chores, caring for children/adults) [54]. Future research should investigate each specific PA domains, such as recreational PA and home duties, when considering mediation analysis.

Overall, effect sizes of direct and indirect effects for neighborhood violence and problems differed slightly. For example, direct effects (path c’) for both neighborhood violence and problems associated with a 3.6 and 3.1 point increase in depressive symptoms, respectively (*p* < .01). In contrast, indirect effects (paths a x b) were statistically significant for neighborhood violence and problems (0.26 and 0.15, respectively), yet the effect sizes were fairly small. Although indirect effects are small, community investment and PA promotion strategies implemented at a community-level could potentially have a large cumulative impact at the population level. Furthermore, our findings of mediation through PA on these associations may have the potential to shed light into the role of PA to reduce the risk for depression for individuals residing in adverse neighborhood environments. Future policy implications may be that, for instance, local public health researchers and practitioners could offer information about PA programs/opportunities to encourage PA in a group setting or at home [55] for those who perceive higher adverse neighborhood social environment.

Our study has several limitations. Given the cross-sectional design, we cannot infer causality. Further studies will be needed to determine the causal relationship by using the longitudinal data of the JHS. Our results may have also been affected by our bootstrapping resampling approach in testing for mediation. Although we could control for clustering effects at the census level due to nesting while investigating our primary aim, we could not account for clustering effects of the neighborhood while performing mediation for the secondary aim. Furthermore, our study sample was limited to the tri-county area of Jackson, MS, so these findings may not generalize to African-American populations in other states or regions of the country. In addition, our neighborhood social environment measures were single, time-invariant measures of the neighborhood environment based on respondents’ perceptions [49]. These were derived from aggregated responses of a self-reported scale, rather than objective data such as police-reported crime data [49]. However, both perceived and objective measures of neighborhood environment are significantly associated with health outcomes [56], which may imply that objective measures are not superior to subjective ones, but rather that both measures have distinct constructs which account for unique variance in health outcomes. It is also possible that our results could be affected by residual confounding if there were unmeasured covariates at the individual or neighborhood level [36, 57]. Because these data came from surveys, they may have been affected by social desirability bias or confidentiality concerns. Though perceived stress (both chronic and weekly stress) was controlled as a confounder to understand the mediating role of physical activity on the association between PNSE and depressive symptoms, it may be that those residing in areas with greater neighborhood violence and problems may be exposed to greater stress with less resources to manage stressors. This may result in greater risk for depression [8]. Lastly, we used self-reported measures of each PNSE, which might lead to the same-source bias [58]. However, these neighborhood measures were created to use empirical Bayes estimates at the census tract level [36]. This procedure has offered more appropriate neighborhood-level measures to minimize measurement errors at individual-level measures [37]. Our study also has several strengths. One major strength is the Jackson Heart Study, a large cohort of African-American participants with particularly rich data given its wide array of psychosocial questionnaires. In addition, our work is novel given its use of mediation testing to investigate the relationship between PNSE and depressive symptoms through PA.

## Conclusions

Our study found that higher levels of neighborhood violence and problems were positively associated with depressive symptoms among JHS participants. Higher levels of PA mediated the associations between neighborhood violence and problems and depressive symptoms. However, direct and indirect associations between social cohesion and depressive symptoms were not significant in this sample of African Americans. Neighborhood level interventions could address features of the social and built environments (e.g., designing better access to parks and public transit, more street lighting, and open space to promote PA [51, 59]), which may alter neighborhood violence and problems and thus alleviate the burden of depression among African Americans.

## Supplementary information

**Additional file 1.** JHS Neighborhood Survey Data.

**Additional file 2: Table S1.** Associations between neighborhood violence and depressive symptoms stratified by age and gender among JHS participants.

**Additional file 3: Table S2.** Associations between neighborhood problems and depressive symptoms stratified by age and gender among JHS participants.

**Additional file 4: Table S3.** Associations between neighborhood social cohesion and depressive symptoms stratified by age and gender among JHS participants.

**Additional file 5: Table S4.** Indirect and direct associations of neighborhood social environment (IV) with depressive symptoms (DV) through mediators (M) in JHS participants (*n* = 2114)^a^.

**Additional file 6: Table S5.** Indirect and direct associations of neighborhood social environment (IV) with depressive symptoms (DV) through active living mediator (M) in JHS participants (*n* = 2209).

**Additional file 7: Table S6.** Indirect and direct associations of neighborhood social environment (IV) with depressive symptoms (DV) through home/life activities mediator (M) in JHS participants (*n* = 2209).

**Additional file 8: Table S7.** Indirect and direct associations of neighborhood social environment (IV) with depressive symptoms (DV) through sport/exercise activities mediator (M) in JHS participants (*n* = 2209).

**Additional file 9: Table S8.** Selected key participants’ characteristics included and not included in analyses.

**Additional file 10: Figure S1.** Associations between neighborhood violence and depressive symptoms score, adjusting for all individual, health-related, psychosocial, and environmental factors, stratified by age and sex.

**Additional file 11: Figure S2.** Associations between neighborhood problems and depressive symptoms score, adjusting for all individual, health-related, psychosocial, and environmental factors, stratified by age and sex.

**Additional file 12: Figure S3.** Associations between neighborhood social cohesion and depressive symptoms score, adjusting for all individual, health-related, psychosocial, and environmental factors, stratified by age and sex.

## Data Availability

The data in the present study came from the Jackson Heart Study (https://www.jacksonheartstudy.org/). These data are not publicly available, and its use is restricted.

## Abbreviations

JHS: Jackson Heart Study
PNSE: Perceived Neighborhood Social Environment
BC CIs: Bias Corrected Confidence Intervals
NHANES: National Health and Nutrition Examination Survey
CES-D: Center for Epidemiological Studies Depression Score
MESA: Multi-Ethnic Study of Atherosclerosis
JPAC: Jackson Heart Study Physical Activity Instrument
