# Supplementary material for: Physical activity-mediated associations between perceived neighborhood social environment and depressive symptoms among Jackson Heart Study participants
Source: Int J Behav Nutr Phys Act. 2020 Jul 10;17:91. doi: 10.1186/s12966-020-00991-y (PMC7350640; doi:10.1186/s12966-020-00991-y)
Supplement: Supplementary file 1 — Additional file 1. JHS Neighborhood Survey Data. [file 12966_2020_991_MOESM1_ESM.docx]

**Supplemental File 1**

### JHS Neighborhood Survey Data

The JHS neighborhood survey data includes variables contextualizing neighborhood social characteristics around JHS participants’ homes. Seventeen survey items below asked the participants to report the conditions of their neighborhood issues related to exposure to violence (4=O: Often to 1=N: Never), neighborhood problems (4=V: Very serious problem to 1=N: Not really a problem), and social cohesion (4=A: Strongly agree to 1=D: Strongly disagree),.

Principal component analysis (PCA) on the 17 items showed that they could be grouped into three variables. However, the item, “This neighborhood is safe from crime.” Was removed due to low factor loading.

All the items used to measure each PNSE (available from: <https://www.jacksonheartstudy.org/Portals/0/pdf/form1/AF3A-F.pdf?ver=2015-07-08-132115-310>)

| **Description** **of perceived neighborhood social environment** | |
| --- | --- |
| Variable | **Item** |
| Neighborhood Violence | How often was there a fight in this neighborhood in which a weapon was used |
|  | How often was there a violent argument between neighbors |
|  | How often were there gang fights? |
|  | How often was there a sexual assault or rape? |
|  | How often was there a robbery or mugging? |
| Neighborhood Problems | Excessive noise |
|  | Heavy traffic or speeding cars |
|  | Lack of access to adequate food and/or shopping |
|  | Lack of parks and playground |
|  | Trash and Litter |
|  | No sidewalks and poorly maintained sidewalks |
| Neighborhood Social cohesion | This is a close knit neighborhood |
|  | People around here are willing to help their neighbors |
|  | People in this neighborhood generally don't get along |
|  | People in this neighborhood can be trusted |
|  | People in this neighborhood do not share the same values |
|  | This neighborhood is safe from crime* |

Note: *”This neighborhood is safe from crime.” was excluded due to low factor loading.
