# Supplementary material for: Physical activity-mediated associations between perceived neighborhood social environment and depressive symptoms among Jackson Heart Study participants
Source: Int J Behav Nutr Phys Act. 2020 Jul 10;17:91. doi: 10.1186/s12966-020-00991-y (PMC7350640; doi:10.1186/s12966-020-00991-y)
Supplement: Supplementary file 2 — Additional file 2: Table S1. Associations between neighborhood violence and depressive symptoms stratified by age and gender among JHS participants. [file 12966_2020_991_MOESM2_ESM.docx]

| **Supplemental Table 1.** Associations between neighborhood violence and depressive symptoms stratified by age and gender among JHS participants | | | | | | | | |
| --- | --- | --- | --- | --- | --- | --- | --- | --- |
|  | **Age < 55; Females (n=812)** | | **Age ≥ 55; Females (n= 606)** | | **Age < 55; Males (n=473)** | | **Age ≥ 55; Males (n=318)** | |
|  | **B (SE)** | **95% C.I.** | **B (SE)** | **95% C.I.** | **B (SE)** | **95% C.I.** | **B (SE)** | **95% C.I.** |
| **Intercept** | 8.61 (3.54)* | 1.58, 15.63 | 7.32 (3.77) | -0.20, 14.83 | 5.64 (3.57) | -1.45, 12.73 | 8.46 (4.47) | -0.48, 17.39 |
| **Neighborhood Violence** | 5.74 (2.46)* | 0.85, 10.63 | 2.21 (2.68) | -3.15, 7.56 | 4.41 (2.38) | -0.33, 9.15 | 0.55 (3.12) | -5.68, 6.77 |
| **Individual Characteristics** |  |  |  |  |  |  |  |  |
| High School Graduate |  |  |  |  |  |  |  |  |
| No | Ref. | |  | | Ref. | |  | |
| Yes | -3.29 (1.29)* | -5.98, -0.59 | -2.23 (0.69)** | -3.63, -0.82 | -0.49 (1.21) | -3.03, 2.04 | -1.80 (0.80)* | -3.45, 0.14 |
| Income |  |  |  |  |  |  |  |  |
| ≥$50,000 | Ref. | |  | | Ref. | |  | |
| <$50,000 | 1.32 (0.58)* | 0.16, 2.48 | 2.34 (0.67)** | 0.97, 3.71 | 1.90 (0.62)** | 0.66, 3.14 | 2.10 (0.75)** | 0.57, 3.63 |
| Not reported | 1.54 (0.81) | -0.09, 3.17 | 1.14 (0.91) | -0.73, 3.01 | 0.65 (0.84) | -1.05, 2.35 | 0.65 (1.16) | -1.79, 3.10 |
| Health-Related Factors |  |  |  |  |  |  |  |  |
| Body Mass Index | 0.03 (0.03) | -0.03, 0.09 | 0.04 (0.04) | -0.04, 0.11 | -0.07 (0.04) | -0.16, 0.01 | -0.01 (0.06) | -0.13, 0.10 |
| Total Physical Activity | -0.49 (0.14)*** | -0.76, -0.22 | -0.07 (0.14) | -0.34, 0.21 | -0.17 (0.14) | -0.44, 0.11 | 0.04 (0.16) | -0.27, 0.35 |
| Current smoker |  |  |  |  |  |  |  |  |
| No | Ref. | |  | | Ref. | |  | |
| Yes | 1.48 (0.86) | -0.25, 3.21 | 0.06 (0.88) | -1.74, 1.87 | 1.15 (0.76) | -0.38, 2.69 | -0.33 (1.07) | -2.58, 1.91 |
| Alcohol drinker |  |  |  |  |  |  |  |  |
| No | Ref. | |  | | Ref. | |  | |
| Yes | -0.51 (0.51) | -1.53, 0.52 | -0.44 (0.58) | -1.61, 0.74 | 1.21 (0.57)* | 0.06, 2.36 | 0.21 (0.64) | -1.10, 1.53 |
| Disabled from walking |  |  |  |  |  |  |  |  |
| No | Ref. | |  | | Ref. | |  | |
| Yes | 4.36 (1.42)** | 1.40, 7.32 | -0.28 (0.98) | -2.33, 1.76 | 2.55 (2.27) | -3.28, 8.38 | 1.14 (1.58) | -2.72, 4.99 |
| History of Medical Condition |  |  |  |  |  |  |  |  |
| No | Ref. | |  | | Ref. | |  | |
| Yes | 1.22 (0.56)* | 0.09, 2.35 | 0.98 (0.53) | -0.09, 2.05 | 0.07 (0.70) | -1.34, 1.48 | 1.43 (0.65)* | 0.10, 2.75 |
| **Psychosocial Factors** |  |  |  |  |  |  |  |  |
| Lifetime discrimination | -0.64 (0.32)* | -1.27, -0.01 | -0.35 (0.35) | -1.04, 0.35 | -0.25 (0.35) | -0.94, 0.44 | -0.53 (0.39) | -1.30, 0.23 |
| Daily discrimination | 0.82 (0.30)** | 0.24, 1.40 | 0.93 (0.32)** | 0.31, 1.56 | 0.90 (0.30)** | 0.30, 1.49 | 0.52 (0.37) | -0.21, 1.24 |
| Burden of lifetime discrimination | 1.20 (0.30)*** | 0.61, 1.80 | 0.75 (0.29)* | 0.18, 1.33 | -0.10 (0.33) | -0.75, 0.56 | 0.62 (0.35) | -0.06, 1.30 |
| Chronic stress | 1.67 (0.29)*** | 1.10, 2.23 | 0.91 (0.31)** | 0.31, 1.51 | 1.17 (0.32)*** | 0.54, 1.79 | 0.53 (0.40) | -0.26, 1.32 |
| Weekly stress | 3.39 (0.27)*** | 2.86, 3.92 | 2.38 (0.30)*** | 1.79, 2.97 | 2.13 (0.28)*** | 1.58, 2.68 | 3.35 (0.43)*** | 2.51, 4.19 |
| **Built Environment** |  |  |  |  |  |  |  |  |
| Population Density | -0.99 (0.58) | -2.13, 0.15 | 0.36 (0.65) | -0.91, 1.63 | -0.09 (0.67) | -1.40, 1.23 | 1.05 (0.75) | -0.43, 2.54 |
| ***Note***: ^a^Neighborhood violence was based on unconditional empirical Bayes estimation adjusting for age and sex. ^b^Based on scale sores which were standardized by computing z scores with mean zero and one standard deviation. ^c^Population density (1000 people/km^2^) was measured around one mile from participant’s residence. P-values: *p<0.05; **p<0.01; ***p<0.001. | | | | | | | | |
