# Supplementary material for: Physical activity-mediated associations between perceived neighborhood social environment and depressive symptoms among Jackson Heart Study participants
Source: Int J Behav Nutr Phys Act. 2020 Jul 10;17:91. doi: 10.1186/s12966-020-00991-y (PMC7350640; doi:10.1186/s12966-020-00991-y)
Supplement: Supplementary file 3 — Additional file 3: Table S2. Associations between neighborhood problems and depressive symptoms stratified by age and gender among JHS participants. [file 12966_2020_991_MOESM3_ESM.docx]

| **Supplemental Table 2**. Associations between neighborhood problems and depressive symptoms stratified by age and gender among JHS participants | | | | | | | | |
| --- | --- | --- | --- | --- | --- | --- | --- | --- |
|  | **Age < 55; Females (n=812)** | | **Age ≥ 55; Females (n= 606)** | | **Age < 55; Males (n=473)** | | **Age ≥ 55; Males (n=318)** | |
|  | **B (SE)** | **95% C.I.** | **B (SE)** | **95% C.I.** | **B (SE)** | **95% C.I.** | **B (SE)** | **95% C.I.** |
| **Intercept** | 8.65 (3.12)** | 2.45, 14.84 | 6.96 (3.35)* | 0.27, 13.65 | 7.06 (3.29)* | 0.53, 13.60 | 6.88 (3.93) | -0.97, 14.73 |
| **Neighborhood Problems** | 4.80 (1.69)** | 1.44, 8.16 | 2.10 (1.91) | -1.71, 5.90 | 2.70 (1.73) | -0.75, 6.14 | 1.53 (2.18) | -2.82, 5.87 |
| **Individual Characteristics** |  |  |  |  |  |  |  |  |
| High School Graduate |  |  |  |  |  |  |  |  |
| No | Ref. | |  | | Ref. | |  | |
| Yes | -3.32 (1.28)* | -6.00, -0.63 | -2.22 (0.69)** | -3.63, -0.82 | -0.56 (1.21) | -3.09, 1.98 | -1.75 (0.80)* | -3.40, -0.10 |
| Income |  |  |  |  |  |  |  |  |
| ≥$50,000 | Ref. | |  | | Ref. | |  | |
| <$50,000 | 1.26 (0.58)* | 0.10, 2.42 | 2.29 (0.68)** | 0.91, 3.67 | 1.90 (0.62)** | 0.65, 3.15 | 2.02 (0.75)* | 0.47, 3.56 |
| Not reported | 1.52 (0.81) | -0.11, 3.14 | 1.13 (0.91) | -0.74, 3.00 | 0.70 (0.84) | -1.00, 2.39 | 0.63 (1.16) | -1.80, 3.07 |
| Health-Related Factors |  |  |  |  |  |  |  |  |
| Body Mass Index | 0.03 (0.03) | -0.04, 0.09 | 0.04 (0.04) | -0.04, 0.11 | -0.07 (0.04) | -0.16, 0.01 | -0.01 (0.06) | -0.12, 0.10 |
| Total Physical Activity | -0.48 (0.14)*** | -0.75, -0.21 | -0.07 (0.14) | -0.34, 0.20 | -0.17 (0.14) | -0.45, 0.11 | 0.04 (0.16) | -0.27, 0.36 |
| Current smoker |  |  |  |  |  |  |  |  |
| No | Ref. | |  | | Ref. | |  | |
| Yes | 1.54 (0.85) | -0.19, 3.26 | 0.05 (0.89) | -1.76, 1.85 | 1.13 (0.76) | -0.40, 2.66 | -0.37 (1.07) | -2.61, 1.88 |
| Alcohol drinker |  |  |  |  |  |  |  |  |
| No | Ref. | |  | | Ref. | |  | |
| Yes | -0.45 (0.51) | -1.47, 0.58 | -0.42 (0.58) | -1.60, 0.75 | 1.23 (0.57)* | 0.09, 2.38 | 0.22 (0.64) | -1.08, 1.53 |
| Disabled from walking |  |  |  |  |  |  |  |  |
| No | Ref. | |  | | Ref. | |  | |
| Yes | 4.32 (1.41)** | 1.37, 7.28 | -0.29 (0.98) | -2.34, 1.75 | 2.47 (2.27) | -3.36, 8.31 | 1.16 (1.57) | -2.68, 5.01 |
| History of Medical Condition |  |  |  |  |  |  |  |  |
| No | Ref. | |  | | Ref. | |  | |
| Yes | 1.24 (0.56)* | 0.12, 2.37 | 0.98 (0.53) | -0.09, 2.05 | 0.06 (0.70) | -1.35, 1.47 | 1.46 (0.65)* | 0.13, 2.78 |
| **Psychosocial Factors** |  |  |  |  |  |  |  |  |
| Lifetime discrimination | -0.63 (0.32)* | -1.26, -0.00 | -0.35 (0.35) | -1.04, 0.35 | -0.23 (0.35) | -0.92, 0.46 | -0.54 (0.39) | -1.30, 0.23 |
| Daily discrimination | 0.81 (0.30)** | 0.23, 1.39 | 0.93 (0.32)** | 0.31, 1.56 | 0.89 (0.30)** | 0.29, 1.48 | 0.50 (0.37) | -0.23, 1.22 |
| Burden of lifetime discrimination | 1.21 (0.30)*** | 0.62, 1.81 | 0.76 (0.29)** | 0.19, 1.34 | -0.11 (0.33) | -0.77, 0.55 | 0.60 (0.34) | -0.08, 1.28 |
| Chronic stress | 1.64 (0.29)*** | 1.08, 2.21 | 0.90 (0.31)** | 0.31, 1.50 | 1.19 (0.32)*** | 0.57, 1.82 | 0.55 (0.40) | -0.24, 1.35 |
| Weekly stress | 3.38 (0.27)*** | 2.86, 3.91 | 2.37 (0.30)*** | 1.78, 2.96 | 2.11 (0.28)*** | 1.57, 2.66 | 3.35 (0.42)*** | 2.52, 4.19 |
| **Built Environment** |  |  |  |  |  |  |  |  |
| Population Density | -1.24 (0.60)* | -2.42, -0.06 | 0.16 (0.69) | -0.34, 0.20 | -0.07 (0.71) | -1.47, 1.32 | 0.80 (0.80) | -0.78, 2.38 |
| ***Note***: ^a^Neighborhood problems was based on unconditional empirical Bayes estimation adjusting for age and sex. ^b^Based on scale sores which were standardized by computing z scores with mean zero and one standard deviation. ^c^Population density (1000 people/km^2^) was measured around one mile from participant’s residence. P-values: *p<0.05; **p<0.01; ***p<0.001. | | | | | | | | |
