# Supplementary material for: Physical activity-mediated associations between perceived neighborhood social environment and depressive symptoms among Jackson Heart Study participants
Source: Int J Behav Nutr Phys Act. 2020 Jul 10;17:91. doi: 10.1186/s12966-020-00991-y (PMC7350640; doi:10.1186/s12966-020-00991-y)
Supplement: Supplementary file 4 — Additional file 4: Table S3. Associations between neighborhood social cohesion and depressive symptoms stratified by age and gender among JHS participants. [file 12966_2020_991_MOESM4_ESM.docx]

| **Supplemental Table 3.** Associations between neighborhood social cohesion and depressive symptoms stratified by age and gender among JHS participants | | | | | | | | |
| --- | --- | --- | --- | --- | --- | --- | --- | --- |
|  | **Age < 55; Females (n=812)** | | **Age ≥ 55; Females (n= 606)** | | **Age < 55; Males (n=473)** | | **Age ≥ 55; Males (n=318)** | |
|  | **B (SE)** | **95% C.I.** | **B (SE)** | **95% C.I.** | **B (SE)** | **95% C.I.** | **B (SE)** | **95% C.I.** |
| **Intercept** | 18.92 (7.98)* | 3.07, 34.76 | 16.07 (7.60)* | 0.91, 31.22 | 24.14 (8.21)** | 7.83, 40.46 | 13.42 (8.56) | -3.68, 30.52 |
| **Neighborhood Social Cohesion** | -1.16 (2.50) | -6.12, 3.80 | -1.97 (2.37) | -6.71, 2.76 | -4.30 (2.57) | -9.41, 0.80 | -1.43 (2.75) | -6.92, 4.06 |
| **Individual Characteristics** |  |  |  |  |  |  |  |  |
| High School Graduate |  |  |  |  |  |  |  |  |
| No | Ref. | |  | | Ref. | |  | |
| Yes | -3.50 (1.29)* | -6.21, -0.79 | -2.25 (0.69)** | -3.65, -0.85 | -0.56 (1.21) | -3.09, 1.97 | -1.75 (0.81)* | -3.41, 0.09 |
| Income |  |  |  |  |  |  |  |  |
| ≥$50,000 | Ref. | |  | | Ref. | |  | |
| <$50,000 | 1.45 (0.58)* | 0.29, 2.61 | 2.35 (0.67)*** | 0.98, 3.72 | 1.97 (0.62)** | 0.73, 3.21 | 2.07 (0.74)** | 0.54, 3.59 |
| Not reported | 1.57 (0.81) | -0.06, 3.21 | 1.19 (0.91) | -0.68, 3.06 | 0.69 (0.84) | -1.01, 2.38 | 0.64 (1.16) | 0.54, 3.60 |
| Health-Related Factors |  |  |  |  |  |  |  |  |
| Body Mass Index | 0.03 (0.03) | -0.03, 0.09 | 0.04 (0.04) | -0.04, 0.11 | -0.07 (0.04) | -0.16, 0.01 | -0.01 (0.06) | -0.13, 0.10 |
| Total Physical Activity | -0.49 (0.14)*** | -0.76, -0.22 | -0.07 (0.14) | -0.35, 0.20 | -0.18 (0.14) | -0.46, 0.09 | 0.04 (0.16) | -0.27, 0.36 |
| Current smoker |  |  |  |  |  |  |  |  |
| No | Ref. | |  | | Ref. | |  | |
| Yes | 1.56 (0.86) | -0.18, 3.29 | 0.07 (0.89) | -1.74, 1.87 | 1.13 (0.76) | -0.40, 2.66 | -0.35 (1.07) | -2.59, 1.90 |
| Alcohol drinker |  |  |  |  |  |  |  |  |
| No | Ref. | |  | | Ref. | |  | |
| Yes | -0.58 (0.52) | -1.60, 0.45 | -0.42 (0.58) | -1.60, 0.75 | 1.22 (0.57)* | 0.07, 2.37 | 0.22 (0.64) | -1.08, 1.53 |
| Disabled from walking |  |  |  |  |  |  |  |  |
| No | Ref. | |  | | Ref. | |  | |
| Yes | 4.22 (1.42)** | 1.25, 7.19 | -0.25 (0.99) | -2.29, 1.80 | 2.61 (2.27) | -3.23, 8.45 | 1.22 (1.58) | -2.66, 5.09 |
| History of Medical Condition |  |  |  |  |  |  |  |  |
| No | Ref. | |  | | Ref. | |  | |
| Yes | 1.29 (0.57)* | 0.16, 2.42 | 0.97 (0.53) | -0.10, 2.04 | 0.07 (0.70) | -1.34, 1.47 | 1.42 (0.64)* | 0.10, 2.74 |
| **Psychosocial Factors** |  |  |  |  |  |  |  |  |
| Lifetime discrimination | -0.70 (0.32)* | -1.33, -0.07 | -0.36 (0.35) | -1.05, 0.34 | -0.25 (0.35) | -0.93, 0.44 | -0.55 (0.39) | -1.31, 0.22 |
| Daily discrimination | 0.84 (0.30)** | 0.26, 1.43 | 0.93 (0.32)** | 0.31, 1.56 | 0.89 (0.31)** | 0.30, 1.49 | 0.51 (0.37) | -0.22, 1.22 |
| Burden of lifetime discrimination | 1.19 (0.30)*** | 0.59, 1.78 | 0.75 (0.29)* | 0.18, 1.33 | -0.06 (0.33) | -0.72, 0.60 | 0.62 (0.34) | -0.06, 1.29 |
| Chronic stress | 1.70 (0.29)*** | 1.13, 2.26 | 0.92 (0.30)** | 0.32, 1.52 | 1.15 (0.32)*** | 0.52, 1.78 | 0.53 (0.40) | -0.27, 1.32 |
| Weekly stress | 3.42 (0.27)*** | 2.89, 3.95 | 2.37 (0.30)*** | 1.79, 2.96 | 2.11 (0.28)*** | 1.56, 2.65 | 3.35 (0.43)*** | 2.51, 4.18 |
| **Built Environment** |  |  |  |  |  |  |  |  |
| Population Density | -0.50 (0.58) | -1.64, 0.63 | 0.42 (0.60) | -0.76, 1.60 | 0.03 (0.67) | -1.28, 1.34 | 1.00 (0.70) | -0.39, 2.38 |
| ***Note***: ^a^Neighborhood social cohesion was based on unconditional empirical Bayes estimation adjusting for age and sex. ^b^Based on scale sores which were standardized by computing z scores with mean zero and one standard deviation. ^c^Population density (1000 people/km^2^) was measured around one mile from participant’s residence. P-values: *p<0.05; **p<0.01; ***p<0.001. | | | | | | | | |
