# Supplementary material for: Physical activity-mediated associations between perceived neighborhood social environment and depressive symptoms among Jackson Heart Study participants
Source: Int J Behav Nutr Phys Act. 2020 Jul 10;17:91. doi: 10.1186/s12966-020-00991-y (PMC7350640; doi:10.1186/s12966-020-00991-y)
Supplement: Supplementary file 5 — Additional file 5: Table S4. Indirect and direct associations of neighborhood social environment (IV) with depressive symptoms (DV) through mediators (M) in JHS participants (n = 2114)a. [file 12966_2020_991_MOESM5_ESM.docx]

| **Supplemental Table 4**. Indirect and direct associations of neighborhood social environment (IV) with depressive symptoms (DV) through mediators (M) in JHS participants (n=2,114)^a^ | | | | | | | | | |
| --- | --- | --- | --- | --- | --- | --- | --- | --- | --- |
|  | Neighborhood Violence | | | Neighborhood Problems | | | Neighborhood Social Cohesion | | |
|  | B | SE | 95% CI | B | SE | 95% CI | B | SE | 95% CI |
| Path a: IV on M | -1.12** | 0.40 | -1.90, -0.35 | -0.67* | 0.28 | -1.22, -0.12 | 0.74 | 0.38 | -0.01, 1.50 |
| Path b: M on DV | -0.25*** | 0.07 | -0.39, -0.10 | -0.24** | 0.07 | -0.39, -0.10 | -0.25*** | 0.08 | -0.40, -0.11 |
| Path c': Direct effect | 3.65** | 1.35 | 1.01, 6.30 | 3.11** | 0.95 | 1.24, 4.98 | -1.87 | 1.31 | -4.45, 0.70 |
| Paths a x b: Indirect effect | 0.27* | 0.13 | 0.06, 0.57⁑ | 0.16 | 0.09 | 0.03, 0.36⁑ | -0.19 | 0.12 | -0.46, 0.00 |
| **Note**: P-values: *p<.05; **p<.01; ***p<.001. ⁑Statistically significant 95% Bias-Corrected Confidence Interval. IV: Independent variables. DV: Dependent variable. M: Mediators. All models were adjusted for covariates. ^a^The analytic sample was based on the removal of those who are disable from walking (n=95). | | | | | | | | | |
