# Supplementary material for: Physical activity-mediated associations between perceived neighborhood social environment and depressive symptoms among Jackson Heart Study participants
Source: Int J Behav Nutr Phys Act. 2020 Jul 10;17:91. doi: 10.1186/s12966-020-00991-y (PMC7350640; doi:10.1186/s12966-020-00991-y)
Supplement: Supplementary file 6 — Additional file 6: Table S5. Indirect and direct associations of neighborhood social environment (IV) with depressive symptoms (DV) through active living mediator (M) in JHS participants (n = 2209). [file 12966_2020_991_MOESM6_ESM.docx]

| **Supplemental Table 5**. Indirect and direct associations of neighborhood social environment (IV) with depressive symptoms (DV) through active living mediator (M) in JHS participants (n=2,209) | | | | | | | | | |
| --- | --- | --- | --- | --- | --- | --- | --- | --- | --- |
|  | Neighborhood Violence | | | Neighborhood Problems | | | Neighborhood Social Cohesion | | |
|  | B | SE | 95% CI | B | SE | 95% CI | B | SE | 95% CI |
| Path a: IV on M | -0.45** | 0.16 | -0.76, -0.13 | -0.33** | 0.11 | -0.56, -0.11 | 0.21 | 0.16 | -0.10, 0.52 |
| Path b: M on DV | -0.50** | 0.18 | -0.85, -0.15 | -0.49** | 0.18 | -0.84, -0.15 | -0.52** | 0.18 | -0.87, -0.17 |
| Path c': Direct effect | 3.63** | 1.35 | 0.98, 6.27 | 3.05** | 0.95 | 1.19, 4.91 | -2.01 | 1.30 | -4.57, 0.54 |
| Paths a x b: Indirect effect | 0.22 | 0.12 | 0.04, 0.50⁑ | 0.16 | 0.08 | 0.03, 0.35⁑ | -0.11 | 0.10 | -0.32, 0.05 |
| **Note**: P-values: *p<.05; **p<.01; ***p<.001. ⁑Statistically significant 95% Bias-Corrected Confidence Interval. IV: Independent variables. DV: Dependent variable. M: Mediators. All models were adjusted for covariates. | | | | | | | | | |
