# Supplementary material for: Physical activity-mediated associations between perceived neighborhood social environment and depressive symptoms among Jackson Heart Study participants
Source: Int J Behav Nutr Phys Act. 2020 Jul 10;17:91. doi: 10.1186/s12966-020-00991-y (PMC7350640; doi:10.1186/s12966-020-00991-y)
Supplement: Supplementary file 7 — Additional file 7: Table S6. Indirect and direct associations of neighborhood social environment (IV) with depressive symptoms (DV) through home/life activities mediator (M) in JHS participants (n = 2209). [file 12966_2020_991_MOESM7_ESM.docx]

| **Supplemental Table 6**. Indirect and direct associations of neighborhood social environment (IV) with depressive symptoms (DV) through home/life activities mediator (M) in JHS participants (n=2,209) | | | | | | | | | |
| --- | --- | --- | --- | --- | --- | --- | --- | --- | --- |
|  | Neighborhood Violence | | | Neighborhood Problems | | | Neighborhood Social Cohesion | | |
|  | B | SE | 95% CI | B | SE | 95% CI | B | SE | 95% CI |
| Path a: IV on M | -0.01 | 0.12 | -0.26, 0.23 | 0.05 | 0.09 | -0.12, 0.22 | 0.01 | 0.12 | -0.23, 0.24 |
| Path b: M on DV | -0.44+ | 0.23 | -0.90, 0.02 | -0.45+ | 0.23 | -0.91, 0.00 | -0.44+ | 0.23 | -0.90, -0.02 |
| Path c': Direct effect | 3.85** | 1.35 | 1.21, 6.49 | 3.23*** | 0.95 | 1.37, 5.09 | -2.12 | 1.30 | -4.67, 0.43 |
| Paths a x b: Indirect effect | 0.01 | 0.06 | -0.13, 0.15 | -0.02 | 0.05 | -0.13, 0.07 | -0.00 | 0.06 | -0.14, 0.14 |
| **Note**: P-values: +p<0.1; *p<.05; **p<.01; ***p<.001. IV: Independent variables. DV: Dependent variable. M: Mediators. All models were adjusted for covariates. | | | | | | | | | |
