# Supplementary material for: Physical activity-mediated associations between perceived neighborhood social environment and depressive symptoms among Jackson Heart Study participants
Source: Int J Behav Nutr Phys Act. 2020 Jul 10;17:91. doi: 10.1186/s12966-020-00991-y (PMC7350640; doi:10.1186/s12966-020-00991-y)
Supplement: Supplementary file 8 — Additional file 8: Table S7. Indirect and direct associations of neighborhood social environment (IV) with depressive symptoms (DV) through sport/exercise activities mediator (M) in JHS participants (n = 2209). [file 12966_2020_991_MOESM8_ESM.docx]

| **Supplemental Table 7**. Indirect and direct associations of neighborhood social environment (IV) with depressive symptoms (DV) through sport/exercise activities mediator (M) in JHS participants (n=2,209) | | | | | | | | | |
| --- | --- | --- | --- | --- | --- | --- | --- | --- | --- |
|  | Neighborhood Violence | | | Neighborhood Problems | | | Neighborhood Social Cohesion | | |
|  | B | SE | 95% CI | B | SE | 95% CI | B | SE | 95% CI |
| Path a: IV on M | -0.65** | 0.25 | -1.14, -0.17 | -0.37* | 0.17 | -0.71, -0.03 | 0.46+ | 0.24 | -0.01, 0.93 |
| Path b: M on DV | -0.25* | 0.12 | -0.47, -0.02 | -0.25* | 0.12 | -0.47, -0.02 | -0.26* | 0.12 | -0.48, -0.03 |
| Path c': Direct effect | 3.69** | 1.35 | 1.05, 6.34 | 3.12** | 0.95 | 1.26, 4.98 | -2.01 | 1.30 | -4.56, 0.55 |
| Paths a x b: Indirect effect | 0.16 | 0.10 | 0.01, 0.38⁑ | 0.09 | 0.06 | -0.01, 0.24 | -0.12 | 0.08 | -0.31, 0.01 |
| **Note**: P-values: +p<0.1; *p<.05; **p<.01; ***p<.001. ⁑ Statistically significant 95% Bias-Corrected Confidence Interval. IV: Independent variables. DV: Dependent variable. M: Mediators. All models were adjusted for covariates. | | | | | | | | | |
