# Supplementary material for: Physical activity-mediated associations between perceived neighborhood social environment and depressive symptoms among Jackson Heart Study participants
Source: Int J Behav Nutr Phys Act. 2020 Jul 10;17:91. doi: 10.1186/s12966-020-00991-y (PMC7350640; doi:10.1186/s12966-020-00991-y)
Supplement: Supplementary file 9 — Additional file 9: Table S8. Selected key participants’ characteristics included and not included in analyses. [file 12966_2020_991_MOESM9_ESM.docx]

| **Supplemental Table 8.** Selected key participants' characteristics included and not included in analyses | | | | |
| --- | --- | --- | --- | --- |
|  | **Included (n=2209)** | **Not included (n=3092)** | ***P-value*^e^** | **Missing^f^** |
| **Individual characteristics** |  |  |  |  |
| Age (years), M (±SD) | 52.64 (±12.20) | 57.31 (±12.95) | <.0001 | 0 |
| Female, n (%) | 1418 (64.19) | 1945 (62.90) | 0.3372 | 0 |
| High School Graduate, n (%) |  |  | <.0001 | 15 |
| Yes | 1959 (88.68) | 2222 (72.21) |  |  |
| No | 250 (11.32) | 855 (27.79) |  |  |
| Income, n (%) |  |  | <.0001 | 0 |
| ≥$50,000 | 783 (35.45) | 749 (24.22) |  |  |
| <$50,000 | 1140 (51.61) | 1831 (59.22) |  |  |
| Not reported | 286 (12.95) | 512 (16.56) |  |  |
| **Health-related factors** |  |  |  |  |
| Body Mass Index, M (±SD) | 31.83 (±7.18) | 31.69 (±7.28) | 0.4904 | 9 |
| Total Physical Activity, M (±SD) | 6.76 (±1.96) | 6.30 (±1.99) | <.0001 | 269 |
| Depressive Symptoms based on CES-D score | 10.79 (±8.05) | 11.32 (±8.18) | 0.0701 | 1889 |
| **Psychosocial factors, M (±SD)** |  |  |  |  |
| Lifetime discrimination | 3.55 (±1.89) | 2.49 (±2.17) | <.0001 | 202 |
| Daily discrimination | 2.20 (±0.99) | 1.99 (±1.02) | <.0001 | 117 |
| Chronic stress | 5.64 (±4.45) | 4.78 (±4.31) | <.0001 | 46 |
| **Neighborhood Environment at the census tract level, M (±SD)** |  |  |  |  |
| *Perceived neighborhood social environment^c^* |  |  |  |  |
| Violence | 1.25 (±0.12) | 1.27 (±0.13) | <.0001 | 11 |
| Problems | 1.55 (±0.19) | 1.58 (±0.19) | <.0001 | 10 |
| Social cohesion | 3.02 (±0.12) | 3.00 (±0.13) | <.0001 | 10 |
| *Objective built environment^d^* |  |  |  |  |
| Population density (people/km^2^) | 791.61 (±487.82) | 840.51 (±490.39) | 0.0003 | 4 |
| ***Note***: ^a^Based on the median. ^b^Alcohol consumption in the past 12 months. ^c^Each perceived social environment variable was aggregated to census-tract level based on unconditional empirical Bayes estimation adjusting for age and sex. ^d^Population density was measured around 1 mile from participant’s residence. ^e^*P*-values were based on t-tests for continuous variables and chi-square tests for categorical variables. ^f^ Missing for data not included in the analyses | | | | |
