# Supplementary material for: Physical activity-mediated associations between perceived neighborhood social environment and depressive symptoms among Jackson Heart Study participants
Source: Int J Behav Nutr Phys Act. 2020 Jul 10;17:91. doi: 10.1186/s12966-020-00991-y (PMC7350640; doi:10.1186/s12966-020-00991-y)
Supplement: Supplementary file 10 — Additional file 10: Figure S1. Associations between neighborhood violence and depressive symptoms score, adjusting for all individual, health-related, psychosocial, and environmental factors, stratified by age and sex. [file 12966_2020_991_MOESM10_ESM.pptx]

## Slide 1
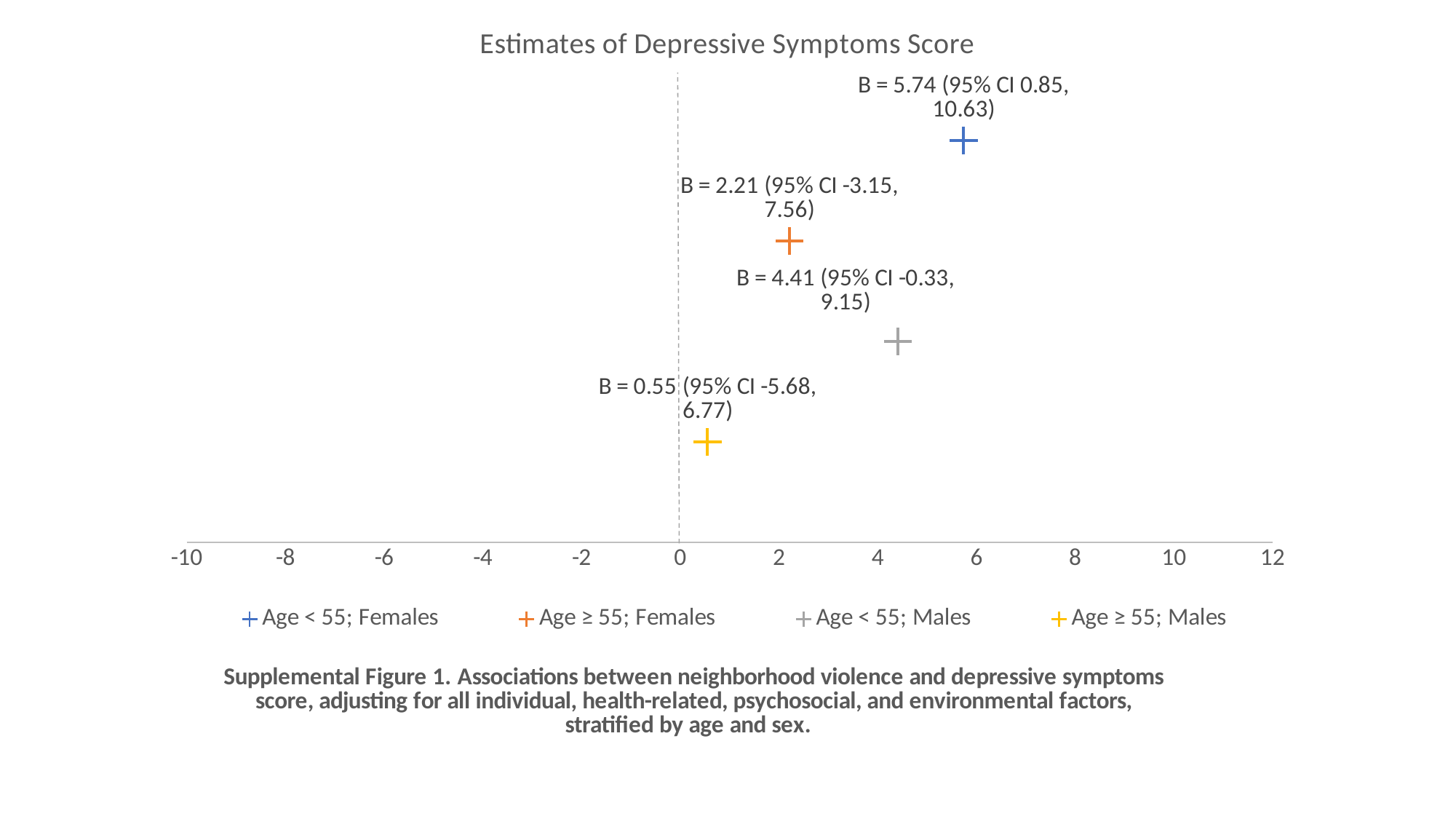

### Chart: Estimates of Depressive Symptoms Score
| Category | Age < 55; Females | Age ≥ 55; Females | Age < 55; Males | Age ≥ 55; Males |
|---|---|---|---|---|
